# The effects of hyperglycemia on brain physiology in a healthy and injured state: An experimental pig study with state-of-the-art multimodal neuromonitoring

**DOI:** 10.1177/0271678X251337633

**Published:** 2025-06-26

**Authors:** Teodor Svedung Wettervik, Anders Hånell, Kerstin M Ahlgren, Henrik Engquist, Anders Lewén

**Affiliations:** 1 Department of Medical Sciences, Section of Neurosurgery, Uppsala University, Uppsala, Sweden; 2 Department of Surgical Sciences, Uppsala University, Uppsala, Sweden

**Keywords:** Brain injury, brain physiology, cerebral blood flow, hyperglycemia, inverse translational, neurointensive care

## Abstract

Although hyperglycemia is associated with worse outcome following acute brain injury, the pathomechanisms remain elusive. In this experimental pig study, we explored the effects of hyperglycemia on brain physiology. Six pigs were anesthetized and received multimodal neuromonitoring of intracranial pressure (ICP), cerebral perfusion pressure (CPP), cerebral autoregulatory metrics (PRx, CBFx, ORx, and with transfer function analysis), cerebral blood flow (CBF), partial brain tissue oxygenation (pbtO_2_), and cerebral microdialysis (MD). The effect of hyperglycemia was studied in the healthy brain after injection of intravenous glucose injections, which increased MD-glucose, while ICP, CPP, PRx, CBFx, ORx pbtO_2_, and cerebral energy metabolism remained unchanged. After normalization of arterial glucose, an intracranial balloon was inflated to increase ICP, followed by an intravenous glucose injection to study the effect of hyperglycemia in the injured brain. The latter induced a significant CBF elevation, but no changes in PRx, pbtO_2_, or cerebral energy metabolism (but a trend towards higher glucose). Hyperglycemia led to favorable short-term effects on cerebral physiology and the immediate increase in arterial glucose that usually follows acute brain injury may be physiologically neuroprotective and the detrimental role of hyperglycemia is more likely related to cellular and molecular pathophysiological mechanisms or merely a confounder.

## Introduction

Hyperglycemia is common and strongly associated with worse outcome in several acute brain injuries including ischemic stroke,^1^ aneurysmal subarachnoid hemorrhage (aSAH),^2,3^ and traumatic brain injury (TBI).^4,5^ To some extent, these findings may be confounded by the link between higher blood glucose and co-morbidities (diabetes mellitus) or more severe systemic and cerebral primary injuries (stress-induced hyperglycemia).^6^ However, it appears likely that high blood glucose, per se, contributes to worse clinical outcome,^4,7–10^ which may entail physiological, cellular, and molecular pathomechanisms.^7^ Taking into account the strong prognostic role of blood glucose in these diseases, it is of great importance to elucidate these pathophysiological pathways in greater detail.

Regarding the effects of hyperglycemia on brain physiology, observational studies on severe acute brain injury patients with multimodal neuromonitoring indicate that higher arterial glucose worsen cerebral pressure autoregulation (higher pressure reactivity index [PRx]) in TBI^4,8^ and predispose for detrimental cerebral ischemia and hyperemia.^11^ Preclinical studies also corroborate that acute hyperglycemia alters the cerebrovascular myogenic and endothelial response and impacts on cerebral blood flow (CBF) levels.^9,12–16^ However, hyperglycemia may be favorable by augmenting cerebral glucose delivery,^17^ particularly in a compromised brain state with poor cerebral substrate supply or impaired energy metabolic turnover.^18,19^ Thus, hyperglycemia may both exert beneficial and detrimental effects on the brain. To increase our understanding on the effect of hyperglycemia on brain physiology and bridge the indicative observations from acute brain injury patients treated in the neurointensive care, this study aimed to investigate the effect of high blood glucose on the brain in a healthy and injured cerebral state. This was done in an experimental pig model using state-of-the-art multimodal neuromonitoring from the clinical setting. We hypothesized that hyperglycemia would impair cerebral pressure autoregulation and increase CBF and cerebral glucose levels, both in the healthy and injured brain state.

## Materials and methods

### Animals and ethic statements

We included six pigs (*Sus scrofa domesticus*, three-breed cross; Norwegian Landrace [1/4], Yorkshire [1/4], and Hampshire [1/2], 3 females and 3 males) aged 2–3 months (Supplemental Table 1). The study was approved by the Animal Ethics Committee in Uppsala, Sweden (Dnr 5.8.18-21799/2022) and performed in compliance with the European Communities Council Directive (2010/63/EU) at the Hedenstierna laboratory, Uppsala University, Sweden. All applicable institutional and national guidelines for the care and use of animals were followed. The study was conducted in compliance with the ARRIVE guidelines 2.0 for the reporting of animal experiments.

### Anesthesia and mechanical ventilation

The pigs were premedicated with Tiletamin/zolazepam 6 mg/kg (Zoletil Forte, Virback, Kolding, Denmark) and xylazine 2.2 mg/kg (Rompun, Elanco Denmark Aps, Ballerup, Denmark) and given a bolus of fentanyl 5 μg/kg (Braun, Danderyd, Sweden) when intravenous access was established. Anesthesia was maintained with ketamine (Abcur, Helsingborg, Sweden) 30 mg/kg/h, fentanyl (Braun, Danderyd, Sweden) 4 μg/kg/h and midazolam (Accord-Healthcarre, Solna, Sweden) 0.12 mg/kg/h during the whole experiment. After adequate levels of anesthesia and analgesia were ascertained by the absence of reaction to pain stimulus between the rear hooves, rocuronium (Braun, Kista, Sweden) 2.5 mg/kg/h was infused intravenously as a muscle relaxant. Ringer acetate (Baxter, Kista, Sweden) was infused intravenously at a rate of 10 mL/kg/h during the first hour, and thereafter at a rate of 5 mL/kg/h. Animals were tracheostomized and mechanically ventilated (Servo I, Maquet, Solna, Sweden).

### Study design

The pigs received multimodal neuromonitoring probes/catheters in the right frontal lobe to study intracranial pressure (ICP), PRx, CBF, partial brain tissue oxygenation (pbtO_2_), and cerebral energy metabolism, in addition to systemic monitoring of arterial blood pressure (ABP) and blood gases (ABG) via an arterial line in the right carotid artery. A pulmonary artery catheter and a triple lumen central venous catheter were inserted via the right jugular vein and used for monitoring the animals and for fluid infusions. Furthermore, an intracranial balloon was inserted in the epidural space in proximity to the frontoparietal lobes via a contralateral burr hole.

**Figure 1.**
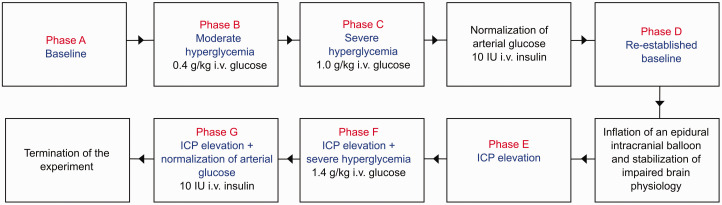
Flowchart of the experimental design. The figure illustrates the experimental design of this pig study. Each phase included 30 minutes of monitoring. ICP: intracranial pressure.

The effect of hyperglycemia on brain physiology was studied both in a healthy and an injured cerebral state (Figure 1). Every physiological manipulation was followed by 30 minutes of physiological monitoring. First, the effect of hyperglycemia on brain physiology in a healthy cerebral state was investigated. After baseline monitoring (*phase A*), a moderate (0.4 g/kg) intravenous bolus dose of glucose was injected (*phase B*). After 30 minutes, severe hyperglycemia was induced by a second and larger (1.0 g/kg) intravenous injection of glucose (*phase C*). Thirty minutes after the second glucose injection, an intravenous insulin dose (around 10 IU) was given to restore normoglycemia. A re-established baseline was then monitored for 30 minutes (*phase D*). Second, to study the effect of hyperglycemia on brain physiology in the injured state, the intracranial balloon was inflated with approximately 5 mL of water to induce intracranial hypertension and cerebral ischemia. The balloon was inflated to lower CPP to approximately 30 mmHg, which is the estimated threshold for cerebral energy metabolic decompensation in these pigs based on previous findings.^20^ The final CPP target was also adapted based on the other neuromonitoring tools, to make sure that the brain state was compromised with reduced CBF and lower pbtO_2_. Once the physiological variables had stabilized at the targeted CPP, monitoring was performed for 30 minutes (*phase E*). Subsequently, a bolus dose (1.4 g/kg) of glucose was injected to induce hyperglycemia (*phase F*). Lastly, insulin (around 10 IU) was administered intravenously to restore arterial glucose during the following 30 minutes (*phase G*) and then the experiment was terminated.

### Monitors, data acquisition, and analysis

ABP was monitored continuously at 100 Hz via an arterial line. ABG was sampled every 15 minutes during each phase and also 5 minutes after the injection of glucose during the hyperglycemic phases. The estimated plasma osmolality was calculated as “2[Na] + [Glucose]”,^21^ i.e., without urea [Urea] in blood (not available) as in the traditional formula. A standard pulse oximeter was attached to the ear to monitor systemic oxygen saturation (SpO_2_). The neuromonitors were inserted via separate burr holes in the right frontal lobe (Supplemental Figure 1). The Neurovent PTO (Raumedic, Germany) was used for continuous monitoring of pbtO_2_ at 1 Hz and ICP at 100 Hz. The thermal diffusion probe (Bowman Perfusion Monitor, Hemedex, USA) monitored regional CBF and brain temperature at 1 Hz, but occasionally required re-calibrations. These data were collected into the Moberg CNS Monitor (USA). Data artefacts, e.g., for ABP during retrieval of ABGs, were removed automatically and manually. PRx was calculated as the Pearson correlation coefficient of 10 s values between ABP and ICP over the last 5 minutes.^22,23^ The oxygen reactivity index (ORx) was calculated as the Pearson correlation coefficient of pbtO_2_ and CPP based on 30 s values over 30 minutes, i.e., shorter than the traditional 60-minute calculations to match the length of each study phase.^24^ We also calculated a similar index (CBFx) based on the Pearson correlation coefficient of CBF and CPP using 30 s values over each 30-minute phase.^25^ Furthermore, transfer function analysis (TFA)^26^ was performed to assess the cerebral autoregulatory status using CBF measurements at 1 Hz and ABP down-sampled to 1 Hz. Intervals were discarded before analysis if CBF data were unavailable due to recalibration of the measuring device or rapidly changing due to glucose administration. Brief ABP artefacts caused by ABG sampling were replaced using linear interpolation. Calculations were performed with the R library clintools (version 0.9.10.1) using the function TFA with default settings. The window length was 102.4 seconds with 59.99% overlap, which resulted in approximately 30 windows for most intervals. Phases, normalized gain, and coherence were evaluated for the very low frequency (VLF; 0.02–0.07 Hz), low frequency (LF; 0.07–0.20 Hz), and high frequency (HF; 0.20–0.50 Hz). A coherence below 0.2 was considered unreliable.

The cerebral MD (M Dialysis AB, Sweden), the 71 High Cut-Off Brain MD catheter, with a membrane length of 10 mm and a membrane cut-off of 100 kDa, was used for energy metabolic monitoring. The catheter was perfused by means of a microinjection pump with a rate at 2 µL/minute using custom made sterile artificial cerebrospinal fluid (NaCl 147 mmol/L (mM), KCl 2.7 mM, CaCl_2_ 1.2 mM, MgCl_2_ 0.85 mM and 1.5% human albumin). The fluid was collected in vials every 15 minutes and immediately put in a −20°C freezer. Cerebral glucose, pyruvate, lactate, glycerol, glutamate, and urea from these vials were later estimated using a ISCUSflex Microdialysis Analyzer (M Dialysis AB). The lactate-pyruvate-ratio (LPR) was also calculated. Urea was used as a reference to determine the reliability of the MD analyses.^27^ The median values of all systemic and cerebral variables were calculated during each phase.

### Statistical analysis

The statistical analyses were conducted in RStudio software (version 2022.12.0).^28^ For the hyperglycemic challenge in a healthy brain state, the difference in median values for the systemic and cerebral variables were compared between *phase A* (baseline) vs. *phase B* (mild hyperglycemia) and *phase A* vs. *phase C* (severe hyperglycemia) using the Wilcoxon test. For the hyperglycemic challenge in the injured brain state, the median values for the same variables were compared between *phase E* (ICP elevation) vs. *phase F* (ICP elevation + hyperglycemia) and *phase F* vs. *phase G* (ICP elevation + restoration of arterial glucose with insulin) using the same statistical test. A p-value <0.05 was considered statistically significant.

## Results

### The effects of hyperglycemia on brain physiology in a healthy cerebral state

**Figure 2.**
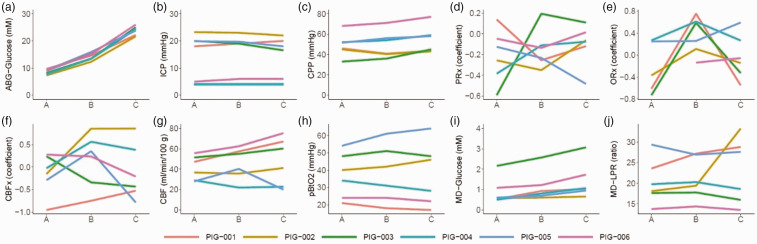
(a–j) Induction of hyperglycemia in the normal brain state – a multimodality monitoring analyses. The figure illustrates the dynamics of the median values during each 30-minute episode of baseline (*phase A*) and following administration of moderate (*phase B*) and large (*phase C*) intravenous glucose injections in six anesthetized pigs with invasive multimodality monitoring (a to j). As illustrated, intravenous glucose injections increased arterial glucose. ICP and CPP remained unchanged, while the PRx response was scattered/heterogenous. CBF increased consistently in four pigs, but not in two cases for whom the monitor re-calibrated during this period. The pbtO_2_ and MD-LPR responses were scattered, while MD-glucose consistently increased with hyperglycemia. The lines in the figures are color-coded for each pig. CBF: cerebral blood flow; CBFx: CBF index; CPP: cerebral perfusion pressure; ICP: intracranial pressure; LPR: lactate-to-pyruvate ratio; MD: microdialysis; ORx: oxygen reactivity index; PbtO_2_: partial brain tissue oxygen; PRx: pressure reactivity index.

**Figure 3.**
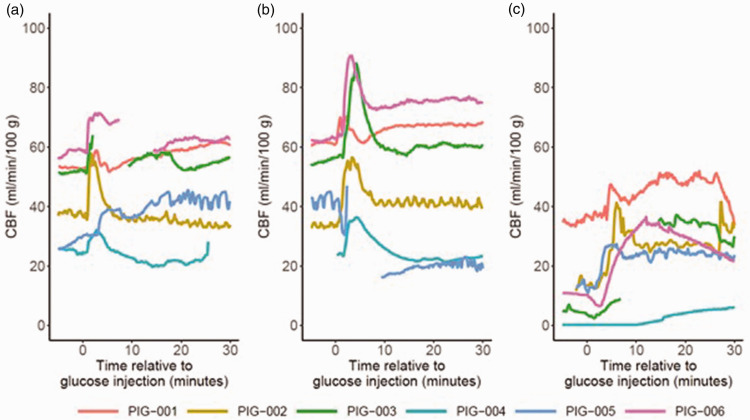
(a–c) Immediate effects of hyperglycemia on CBF. Figure a illustrates that there was a consistent, and usually transient, increase in CBF in all pigs following moderate hyperglycemia (phase B). Similar immediate effects were seen after severe hyperglycemia (phase C, figure b) and after induction of hyperglycemia in case of ICP elevation (phase F, figure c). CBF: cerebral blood flow; ICP: intracranial pressure.

At baseline (Table 1 and Figure 2 and Supplemental Figure 2 and 3), the median arterial glucose was 8.6 (IQR 7.9–9.1) mM, which increased to 14.1 (IQR 13.4–15.0) mM after a mild intravenous glucose injection and to 24.1 (IQR 22.6–24.8) mM after a large intravenous glucose injection (both p < 0.05). While the arterial sodium levels decreased (p < 0.05) during mild and severe hyperglycemia (Supplemental Table 2 and Supplemental Figure 2 and 3), the estimated osmolality remained unchanged. ICP, CPP, PRx, ORx, and CBFx were unaffected during mild and severe hyperglycemia. CBF increased in four of six pigs during both hyperglycemia phases, but in two pigs the monitor was unstable and re-calibrated during these periods. As outlined in Figure 3, all pigs exhibited immediate peaks in CBF that usually were sustained for approximately 10–15 minutes. The TFA analyses revealed relatively higher phase shift in VLF compared to LF and HF ranges and slight dynamic changes in these variables, but the coherence was overall low, particularly for the VLF and LF (<0.20; Supplemental Figure 4 and 5). PbtO_2_ and MD-LPR remained unchanged during hyperglycemia on group level, whereas MD-glucose increased from 0.6 (IQR 0.5–1.0) mM at baseline to 0.9 (IQR 0.7–1.1) mM during mild hyperglycemia and 1.1 (IQR 1.0–1.6) mM during severe hyperglycemia (both p < 0.05). MD-pyruvate, -lactate, -glycerol, and -glutamate were unaffected during hyperglycemia.

**Table 1.** Physiological changes during hyperglycemia in the healthy brain state.

Variables	Baseline(phase A)	Moderate hyperglycemia(phase B)	Severe hyperglycemia(phase C)
*Arterial blood gases*			
pCO_2_ (kPa)	4.6 (4.2–4.9)	4.7 (4.4–4.8)	4.8 (4.7–4.9)
Hemoglobin (g/L)	86 (83–93)	***82 (79–89)^a^***	***80 (77–86)^a^***
Sodium (mM)	137 (135–138)	***134 (133–135)^a^***	***130 (129–131)^a^***
Glucose (mM)	8.6 (7.9–9.1)	***14.1 (13.4–15.0)^a^***	***24.1 (22.6–24.8)^a^***
Lactate (mM)	2.4 (1.5–2.8)	2.5 (1.8–3.1)	2.4 (1.8–2.9)
Estimated osmolality (mmol/kg)	282 (277–285)	282 (280–285)	284 (281 (287)
*Systemic physiology*			
MAP (mmHg)	67 (58–71)	62 (59–73)	64 (63–73)
SpO_2_ (%)	100 (98–100)	99 (98–100)	98 (97–100)
*Cerebral physiology*			
ICP (mmHg)	19 (8–20)	19 (9–20)	17 (9–20)
CPP (mmHg)	49 (45–52)	47 (40–56)	52 (44–59)
PRx (coefficient)	−0.19 (−0.35–−0.07)	−0.18 (−0.25–−0.12)	−0.07 (−0.11–0.00)
ORx (coefficient)	−0.37 (−0.61–0.25)	0.42 (0.15–0.60)	−0.10 (−0.28–0.19)
CBFx (coefficient)	−0.09 (−0.26–0.17)	0.29 (−0.20–0.51)	−0.32 (−0.50–0.23)
CBF (mL/100 g/minute)	42 (31–50)	47 (37–57)	51 (27–66)
PbtO_2_ (mmHg)	37 (27–46)	37 (26–49)	37 (24–48)
MD-glucose (mM)	0.6 (0.5–1.0)	***0.9 (0.7–1.1)^a^***	***1.1 (1.0–1.6)^a^***
MD-pyruvate (µM)	29 (18–36)	30 (19–31)	27 (18–32)
MD-lactate (mM)	0.5 (0.5–0.7)	0.5 (0.4–0.8)	0.5 (0.4–0.7)
MD-LPR (ratio)	19 (18–23)	20 (18–25)	23 (17–29)
MD-glutamate (µM)	4 (2–5)	4 (3–4)	3 (2–4)
MD-glycerol (µM)	11 (9–12)	9 (6–17)	7 (6–9)
Temperature (°C)	37.8 (37.4–37.8)	37.4 (37.2–37.7)	37.1 (36.8–37.7)

The data are presented as medians (IQR). ^a^p < 0.05. Bold ant italics indicate statistical significance. Moderate (*phase B*) and severe (*phase* C) hyperglycemia were tested against the baseline (*phase A*).

CBF: cerebral blood flow; CBFx: CBF index; CPP: cerebral perfusion pressure; ICP: intracranial pressure; LPR: lactate-to-pyruvate ratio; MAP: mean arterial blood pressure; MD: microdialysis; ORx: oxygen reactivity index; PbtO_2_: partial brain tissue oxygen; pCO_2_: partial pressure of carbon dioxide; PRx: pressure reactivity index; SpO_2_: oxygen saturation.

### The effects of hyperglycemia on brain physiology in a compromised cerebral state

**Figure 4.**
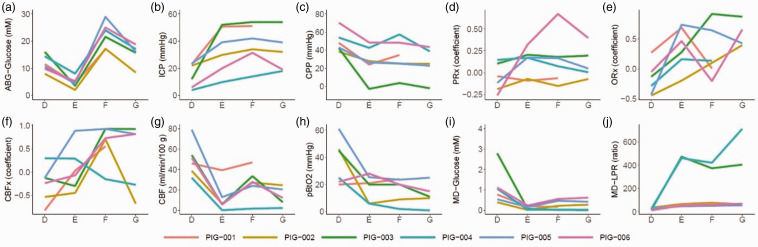
Induction of intracranial hypertension, hyperglycemia, and restoration of arterial glucose in the injured brain state – a multimodality monitoring analyses. The figure illustrates the dynamics of the median values during each 30-minute episode of the re-established baseline (*phase D*), induction of intracranial hypertension with an intracranial, inflatable balloon (*phase* E), administration of a large intravenous glucose injection (*phase* F), and restoration of arterial glucose with insulin (*phase* G) in six anesthetized pigs with invasive multimodality monitoring (a to j). As illustrated, intracranial hypertension compromised the brain state by lowering CPP, increasing PRx, decreasing CBF, pbtO_2_, and MD-glucose, and increasing MD-LPR. Hyperglycemia (*phase* F*)* restored CBF and MD-glucose to some extent, while restoration of arterial glucose with insulin (*phase G*) had the opposite effect. The lines in the figures are color-coded for each pig. CBF: cerebral blood flow; CBFx: CBF index; CPP: cerebral perfusion pressure; ICP: intracranial pressure; LPR: lactate-to-pyruvate ratio; MD: microdialysis; ORx: oxygen reactivity index; PbtO_2_: partial brain tissue oxygen; PRx: pressure reactivity index.

After re-establishing a new baseline following the previous hyperglycemia challenge, a compromised brain state was induced by inflation of an intracranial balloon. After 30 minutes of steady state, a large intravenous glucose bolus was given, which resulted in significantly higher glucose levels from a median of 4.4 (IQR 3.8–5.0) to 22.8 (IQR 18.3–24.8) mM (Table 2 and Figure 4 and Supplemental Figure 6 and 7). At the same time (Supplemental Table 3 and Supplemental Figure 6 and 7), sodium decreased from 135 (IQR 135–137) mM to 129 (IQR 129–130) mM and the estimated osmolality increased from 277 (IQR 274–278) to 280 (IQR 278–286; both p < 0.05). MAP increased during the hyperglycemia challenge (p < 0.05) and, therefore, the balloon was inflated to increase ICP (p < 0.05) in order to keep CPP stable around 30 mmHg (p > 0.05). PRx, and ORx, remained unchanged, while CBF increased significantly from 6 (IQR 6–11) mL/100 g/minute to 28 (25–32) mL/100 g/minute. There was also a trend towards higher CBFx (p = 0.09). Again, the TFA analyses revealed relatively higher phase shift in VLF compared to LF and HF ranges in the compromised cerebral state with slight dynamic changes in these variables, but the coherence was overall low, for the VLF and LF frequencies (<0.20; Supplemental Figure 4 and 5). Furthermore, the CBF improvement did not translate into higher pbtO_2_ or significant changes in the MD-variables. Restoration of arterial glucose with insulin injection did not result in any significant changes in cerebral physiology.

**Table 2.** Physiological changes during hyperglycemia in the injured brain state.

Variables	Baseline(phase D)	High ICP(phase E)	High ICP + hyperglycemia(phase F)	High ICP + insulin(phase G)
*Arterial blood gases*				
pCO_2_ (kPa)	5.2 (5.1–5.3)	5.2 (5.1–5.3)	5.4 (5.2–5.5)	5.4 (5.1–5.5)
Hemoglobin (g/L)	83 (80–91)	83 (79–89)	***79 (72–82)^a^***	77 (75–87)
Sodium (mM)	133 (133–136)	135 (135–137)	***129 (129–130)^a^***	131 (131–133)
Glucose (mM)	11.1 (10.1–13.7)	4.4 (3.8–5.0)	***22.8 (18.3–24.8)^a^***	16.1 (15.8–17.1)
Lactate (mM)	2.6 (2.5–3.1)	1.8 (1.5–2.6)	1.5 (1.3–1.9)	2.0 (1.8–2.0)
Estimated osmolality (mmol/kg)	281 (278–284)	277 (274–278)	***280 (278–286)^a^***	278 (276–281)
*Systemic physiology*				
MAP (mmHg)	63 (59–70)	62 (54–68)	***72 (61–80)^a^***	*57 (54–60)*
SpO_2_ (%)	100 (98–100)	100 (100–100)	100 (99–100)	100 (99–100)
*Cerebral physiology*				
ICP (mmHg)	17 (8–23)	35 (22–48)	***38 (32–49)^b^***	32 (19–39)
CPP (mmHg)	46 (43–53)	27 (24–39)	30 (25–45)	25 (23–39)
PRx (coefficient)	−0.08 (−0.17–0.07)	0.17 (−0.01–0.20)	0.12 (−0.03–0.18)	0.05 (0.00–0.19)
ORx (coefficient)	−0.21 (−0.39- −0.07)	0.37 (0.19–0.63)	0.11 (0.03–0.52)	0.54 (0.42–0.71)
CBFx (coefficient)	−0.18 (−0.46- −0.13)	−0.02 (−0.24–0.22)	0.72 (0.59–0.88)	0.82 (−0.27–0.82)
CBF (mL/100 g/minute)	49 (41–54)	6 (6–11)	***28 (25–32)^a^***	12 (8–20)
PbtO_2_ (mmHg)	35 (23–46)	21 (10–24)	20 (12–23)	11 (10–15)
MD-glucose (mM)	0.9 (0.6–1.1)	0.1 (0.0–0.2)	0.2 (0.1–0.4)	0.3 (0.0–0.4)
MD-pyruvate (µM)	26 (16–34)	9 (5–13)	9 (4–15)	10 (2–15)
MD-lactate (mM)	0.5 (0.4–0.7)	1.0 (0.6–1.3)	0.9 (0.8–1.0)	1.0 (0.8–1.0)
MD-LPR (ratio)	27 (18–29)	66 (58–363)	77 (65–300)	71 (66–406)
MD-glutamate (µM)	2 (2–4)	4 (4–51)	7 (2–44)	7 (2–56)
MD-glycerol (µM)	9 (4–13)	15 (14–28)	17 (8–28)	19 (5–33)
Temperature (°C)	37.0 (36.7–38.0)	36.4 (36.1–38.0)	36.4 (36.1–38.3)	36.3 (36.1–36.7)

The data are presented as medians (IQR). ^a^p < 0.05. Bold ant italics indicate statistical significance. High ICP + hyperglycemia (*phase F*) was tested against high ICP (*phase E*) and high ICP + insulin (*phase G*) against *phase F*.

CBF: cerebral blood flow; CBFx: CBF index; CPP: cerebral perfusion pressure; ICP: intracranial pressure; LPR: lactate-to-pyruvate ratio; MAP: mean arterial blood pressure; MD: microdialysis; ORx: oxygen reactivity index; PbtO_2_: partial brain tissue oxygen; pCO_2_: partial pressure of carbon dioxide; PRx: pressure reactivity index; SpO_2_: oxygen saturation.

## Discussion

In this experimental pig study, induction of hyperglycemia increased CBF. This effect was not explained by changes in CPP or cerebral pressure autoregulation, but was more likely related to hyperglycemia-induced cerebral vasodilation, improved blood rheology, and increased cardiac output. The combination of higher CBF and arterial glucose content (CaGlc) also resulted in higher MD-glucose. Altogether, this rapid and relatively short hyperglycemia period induced favorable short-term effects on brain physiology, which imply that the immediate increase in arterial glucose that usually follows acute brain injury could be physiologically neuroprotective. However, early hyperglycemia is consistently linked with worse outcome in acute brain injury and it is likely that its pathophysiology is rather explained by detrimental cellular and molecular mechanisms.

### The effect of arterial glucose on cerebral blood flow

The main finding of this study was that induction of hyperglycemia led to increased CBF, which was more pronounced in the injured than the healthy state. The thermal diffusion probe re-calibrated during the CBF measurements in the two pigs without any increase in median CBF during hyperglycemia in the healthy brain state. Re-calibration typically alters the baseline CBF to some extent,^29^ which contributed to unreliable comparisons in relation to baseline in these two cases. Otherwise, we would likely have found a significant increase in CBF during hyperglycemia in this scenario, which was consistently demonstrated in the more granular visualization of the CBF dynamics after glucose injection (Figure 2). However, it is also possible that the CBF increase was more amplified by hyperglycemia to restore normal values when the brain was compromised by ischemia, as was found in this study. On the contrary, in the healthy brain state with normal baseline variables, factors that potentially augment CBF may be more counteracted by other mechanisms to avoid hyperemia and maintain homeostasis. Furthermore, there was a clear, but non-significant decrease in CBF in phase G, after administration of insulin during ICP elevation. The decrease in CBF could both be explained by the parallel decrease in arterial glucose and centrally mediated effects by insulin.^30^ However, it seems that the role of insulin on CBF is complex. Particularly, while hypoglycemia was not the focus of this study and insulin was used to restore normoglycemia, McManus et al.^31^ found that CBF increased in case of insulin-mediated hypoglycemia, most likely as a response to preserve cerebral glucose delivery when the arterial content of this metabolite was low. Thus, the net effect of insulin on CBF may depend on the concurrent glucose levels in the blood.

Overall, previous preclinical studies are consistent that hyperglycemia interacts with the regulation of and influences the actual level of CBF.^9,12–16^ The exact mechanisms remain elusive and hyperglycemia have both been shown to decrease^14,15^ and increase^13,16^ CBF in experimental settings in previous studies. Most consistently, and in line with our findings, hyperglycemia seem to decrease the cerebrovascular resistance, possibly by endothelial mechanisms,^13^ and increase CBF.^12,13,16^ In the sections below, we discuss the potential interplay among cerebral autoregulation, CPP, cardiac output, osmolality, and blood rheology in relation to hyperglycemia and CBF.

### The effect of arterial glucose on cerebral autoregulation and perfusion pressure

In contrast to the indicative observations from the neurointensive care, which have found an association between higher arterial glucose and higher PRx in TBI,^4,8^ hyperglycemia did not impact on PRx in this experimental pig study. The reason for the discrepant results may be potential confounding variables such as co-morbidities and injury severity that were not sufficiently addressed in the retrospective clinical studies^4,8^ as compared to our experimental design. On the other hand, the limited amount of monitoring time (30 minutes/phase) and study subjects (n = 6) might have reduced our chance to detect a change in PRx, considering its low signal-to-noise ratio.^32^ Since PRx is expected to change with CPP in a U-shaped way,^33^ similar to the Lassen curve,^11,33^ the effect on the autoregulatory curve may depend on the shape of the curve and the concurrent CPP.^34^ In the healthy state, hyperglycemia per se, may not have been sufficient to impair to autoregulatory status or displace the curve, whereas most pigs were below the lower limit of autoregulation in the compromised brain state, reducing any additional detrimental effect of hyperglycemia. However, PRx is based on ABP and ICP and is a noisy surrogate measure of cerebral autoregulation.^32^ On the contrary, CBFx,^25^ which is a less explored metric based on the actual autoregulatory correlates (CPP and CBF) and would be expected to exhibit a higher signal-to-noise ratio than PRx, showed a clear trend towards higher values during hyperglycemia. This fact still gives some support to the idea^4,8^ that hyperglycemia has a negative effect on cerebral autoregulation.

CPP did not explain the change in CBF, since this variable remained unaffected after the onset of hyperglycemia, both in the normal and injured brain state. Although MAP increased during hyperglycemia in the state of intracranial hypertension, the intracranial balloon was adapted somewhat to keep CPP stable. Another explanation is that hyperglycemia led to higher chronotropy and inotropy to augment cardiac output^35^ and ultimately CBF, however we have no definitive data on cardiac output.

### The effect of arterial glucose on osmolality and blood rheology

It is also possible that the hyperglycemia-related increase in osmolality contributed to cerebrovascular dilation to some extent.^36^ However, the CBF increase during hyperglycemia was most pronounced during the comprised brain state with cerebral ischemia, i.e., when compensatory vasodilation was expected to be near maximum. Even if there may be some vasodilatory reserve left when ischemia has started to occur,^37^ it may be limited, and it therefore appears plausible that other mechanisms were more influential on the CBF increase. Particularly, in parallel with the induction of hyperglycemia, there was a decrease in hemoglobin (hemodilution and hypervolemia), which likely contributed to improved blood rheology or increased circulating volume leading to higher CBF.^38^

### The effect of arterial glucose on brain tissue oxygenation and cerebral energy metabolism

Induction of hyperglycemia led to higher MD-glucose, which was more pronounced in the healthy than the injured brain state. This finding was expected, since the absolute increase in cerebral delivery of glucose (CaGlc * CBF) for the same CaGlc is lower when CBF is compromised. Furthermore, acute brain injury may induce early hypermetabolism,^39^ meaning that the cerebral tissue levels of glucose may remain low despite an increased delivery due to increased consumption. Still, the hyperglycemia-induced increase in CBF and MD-glucose did not translate into down-stream improvements of cerebral oxygenation (pbtO_2_), oxidative energy metabolism (LPR), or neuronal survival (glycerol). In the healthy state, this fact could be explained by that the blood-brain tissue oxygen gradient was already saturated, which may impede further pbtO_2_ increases,^40,41^ while the cerebral energy metabolism was already normal and therefore unaffected by increased MD-glucose supply. In the injured brain state, intracranial hypertension had already occurred for at least 30 minutes, and we speculate whether permanent brain injury and mitochondrial dysfunction impeded recovery of oxygen energy metabolism.^42^

### What is the net effect of acute hyperglycemia on the brain?

While we found that acute hyperglycemia resulted in some favorable short-term effects on brain physiology, other mechanisms may explain the consistent link between hyperglycemia and worse neurological outcome.^2,4,5,10^ These may include detrimental interactions between high arterial glucose and disturbances in coagulation, mitochondrial function, neuroinflammation, and ROS.^7^ Thus, in the clinical setting, although rapid and transient hyperglycemia may ameliorate cerebral ischemia and low MD-glucose, the net effect on the brain of more longstanding hyperglycemia may still be negative due to the aforementioned pathomechanisms.^7,10^

### Methodological consideration

This study had many strengths. The experimental design allowed for more accurate causal inference of the results compared to clinical retrospective studies. The study was based on pigs rather than small, lissencephalic animals, making our findings more translatable to humans. Furthermore, a complete set of clinical state-of-the art multimodal neuromonitoring tools was used, which allowed for in-depth physiological analyses and also increased the direct translatability of our study to the clinical setting.

There were also some limitations. The study was based on only six pigs, increasing the risk for type I- and II-errors. However, the controlled, experimental setting probably contributed to reduce physiological “noise”. We also reported median values and used non-parametric statistical tests, to reduce the effect of influential outliers. Thus, we think that the significant findings in this study were robust, strong, and credible, but a larger cohort might have allowed us to detect also physiological associations with weaker strength. The main source of error was attributed to issues with the neuromonitoring tools. Most particularly, the thermal diffusion probe re-calibrated occasionally, which led to slight shifts in baseline CBF.^29^ This was most notable in two of the pigs during the hyperglycemia challenge in the healthy brain state. Furthermore, although the experiments were started 1–2 hours after insertion of the monitors, it may take longer time (up to 8 hours) before the Neurovent PTO probe has equilibrated,^43^ reducing the reliability of these measurements. Furthermore, during the injured brain state, the inflated volume of the intracranial balloon was repeatedly adjusted due to variations in ABP in order to maintain the targeted CPP. These ICP manipulations may have influenced the validity of PRx as an indicator of autoregulatory function based on spontaneous ICP/ABP fluctuations.^22^ Furthermore, each phase was studied over 30 minutes and therefore reflects the short-term effect of the physiological manipulations, however, we cannot exclude that these effects to change over a longer time interval. Also, data on cardiac output would have contributed to the understanding on the effect of hyperglycemia on brain physiology. However, we decided to avoid additional tools such as the pulse contour cardiac output (PiCCO) monitoring as it would require repeated injections of cold fluid, which could itself influence systemic physiology and further dilute the pigs. In addition, while this study focused on the effects of hyperglycemia on brain physiology, inflammatory protein biomarker profiling from cerebral MD and neurohistological analyses would have added more insights into the cellular and molecular effects of high arterial glucose levels on the brain. Furthermore, the study included both male and female pigs. Although all pigs seemed to demonstrate similar physiological reactions to the hyperglycemic and ICP challenges, we cannot exclude more subtle sex-related differences. However, the fact that the pigs were only 2–3 months and not sexually mature might have attenuated any sex-related difference. Lastly, a TFA was performed based on ABP and CBF (Hemedex probe), rather than CBF velocity (CBFv) from transcranial Doppler (TCD). The use of absolute CBF instead of CBFv may have influenced the frequency-domain analysis, as CBF reflects tissue perfusion rather than arterial flow velocity, potentially introducing additional local metabolic influences. Moreover, the observed low coherence (<0.20) was likely partly attributable to limited ABP variability, which reduces the reliability of TFA-derived metrics. Due to the low coherence, we refrained from performing statistical significance testing on the TFA parameters, as they may not accurately reflect pressure-flow dynamics under these conditions.

## Conclusions

Induction of hyperglycemia increased CBF. This effect was not explained by changes in CPP or cerebral pressure autoregulation, but was more likely related to hyperglycemia-induced cerebral vasodilation, improved blood rheology, and increased cardiac output. The combination of higher CBF and CaGlc also resulted in higher MD-glucose. Altogether, hyperglycemia induced favorable short-term effects on brain physiology, which imply that the immediate increase in arterial glucose that usually follows acute brain injury may be physiologically neuroprotective. However, early hyperglycemia is consistently linked with worse outcome in acute brain injury and it is likely that its pathophysiology is rather explained by detrimental cellular and molecular mechanisms.

## Supplemental Material

sj-pdf-1-jcb-10.1177_0271678X251337633 - Supplemental material for The effects of hyperglycemia on brain physiology in a healthy and injured state: An experimental pig study with state-of-the-art multimodal neuromonitoring Supplemental material, sj-pdf-1-jcb-10.1177_0271678X251337633 for The effects of hyperglycemia on brain physiology in a healthy and injured state: An experimental pig study with state-of-the-art multimodal neuromonitoring by Teodor Svedung Wettervik, Anders Hånell, Kerstin M Ahlgren, Henrik Engquist and Anders Lewén in Journal of Cerebral Blood Flow & Metabolism
