# Supplementary material for: The effects of hyperglycemia on brain physiology in a healthy and injured state: An experimental pig study with state-of-the-art multimodal neuromonitoring
Source: J Cereb Blood Flow Metab. 2025 Jun 26;45(11):2245–55. doi: 10.1177/0271678X251337633 (PMC12202387; doi:10.1177/0271678X251337633)
Supplement: sj-pdf-1-jcb-10.1177_0271678X251337633 - Supplemental material for The effects of hyperglycemia on brain physiology in a healthy and injured state: An experimental pig study with state-of-the-art multimodal neuromonitoring [file sj-pdf-1-jcb-10.1177_0271678X251337633.pdf]

Supplementary table 1. Pig characteristics

Pig	Species	Sex (female/male)	Age (months)	Weight (kg)
1	Sus scrofa domesticus	Male	2-3	31
2	Sus scrofa domesticus	Male	2-3	30
3	Sus scrofa domesticus	Female	2-3	34
4	Sus scrofa domesticus	Female	2-3	31
5	Sus scrofa domesticus	Female	2-3	35
6	Sus scrofa domesticus	Male	2-3	31

Supplementary figure 1. Experimental setting.

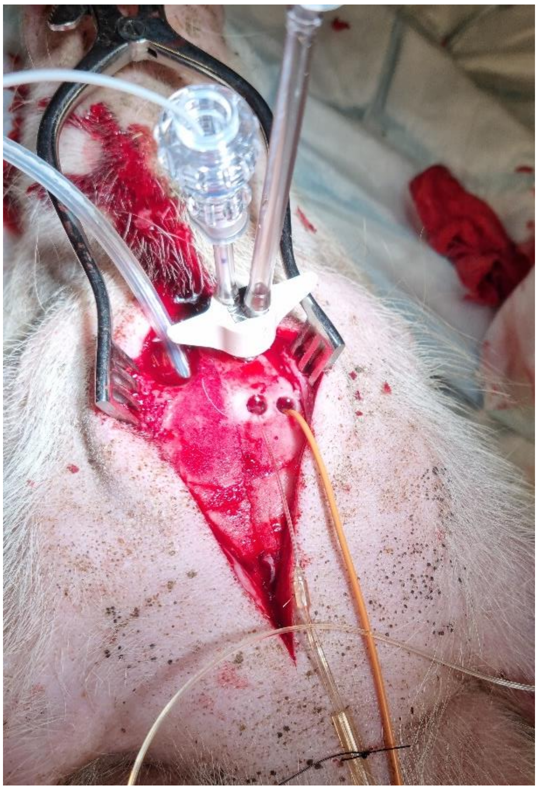

The figure visualizes an anesthetized pig with four separate burr holes in the frontal bone. The right anterior hole contains a bolt with the thermal diffusion probe of CBF fixed in the brain parenchyma. Posteriorly, on the right side, the Neurovent PTO (orange color) with monitoring of ICP and pbtO_2 has been inserted and slightly medial to this monitor, the MD catheter with monitoring of cerebral energy metabolism has been inserted in the brain parenchyma, both via separate burr holes. On the left side, a slightly larger burr hole was made and a catheter with an inflatable balloon was placed epidurally.

CBF = Cerebral blood flow. ICP = Intracranial pressure. MD = Microdialysis. PbtO_2 = Partial brain tissue oxygenation.

Supplementary figure 2. Induction of hyperglycemia in the normal brain state – a multimodality monitoring analyses

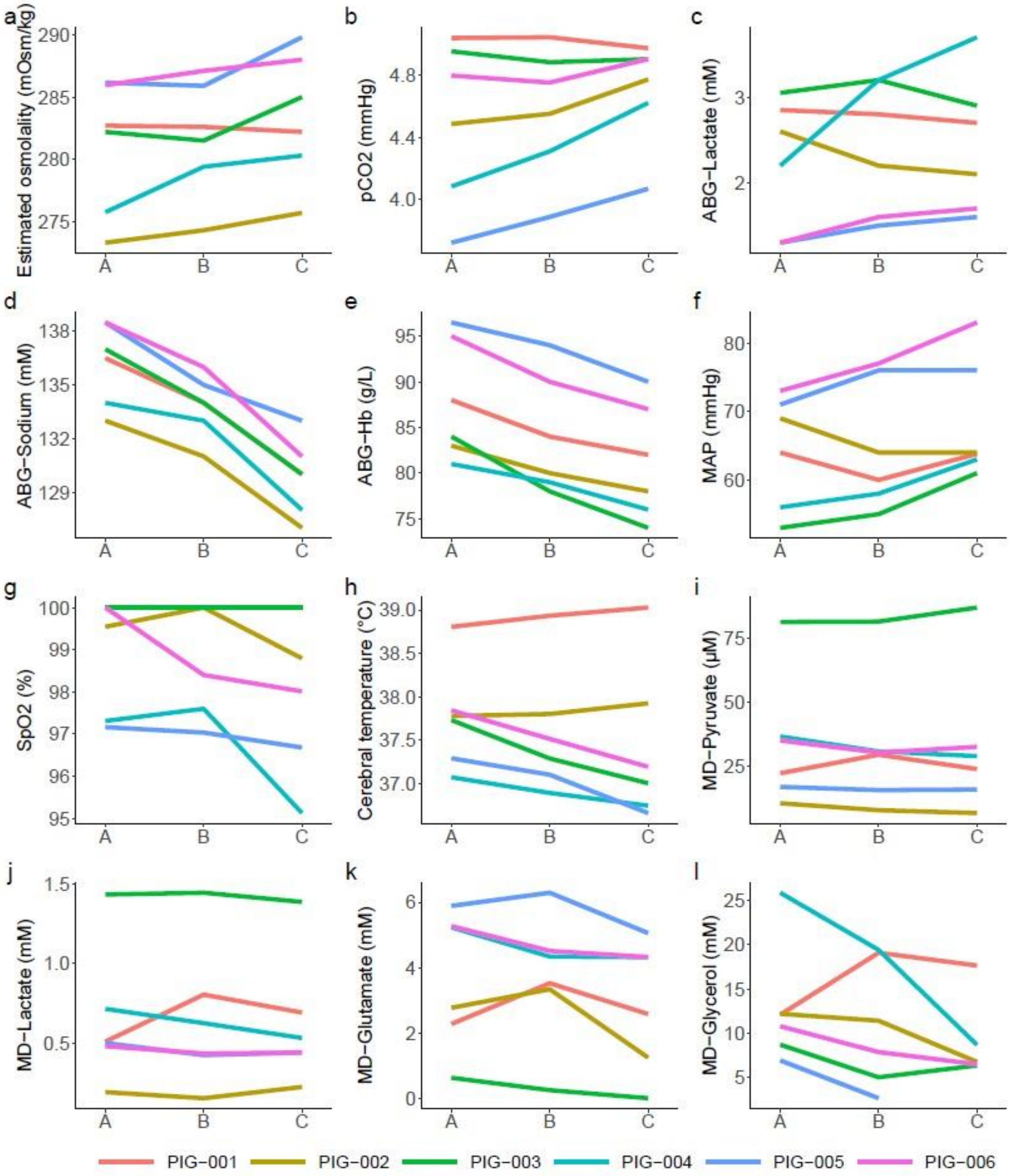

The figure illustrates the dynamics of the median values during each 30-minute episode of baseline (*phase A*) and following administration of moderate (*phase B*) and large (*phase C*) intravenous glucose injections in six anesthetized pigs with invasive multimodality monitoring. As illustrated, intravenous glucose injections increased arterial glucose. ICP and CPP remained unchanged, while the PRx

response was scattered/heterogenous. CBF increased consistently in four pigs, but not in two cases for whom the monitor re-calibrated during this period. The $pbtO_2$ and MD-LPR responses were scattered, while MD-glucose consistently increased with hyperglycemia. The lines in the figures are color-coded for each pig.

CBF = Cerebral blood flow. CPP = Cerebral perfusion pressure. ICP = Intracranial pressure. LPR = Lactate-/pyruvate ratio. MAP = Mean arterial blood pressure. MD = Microdialysis. $PbtO_2$ = Partial brain tissue oxygenation. PRx = Pressure reactivity index.

Supplementary figure 3. Induction of hyperglycemia in the normal brain state – a multimodality monitoring analyses – relative changes

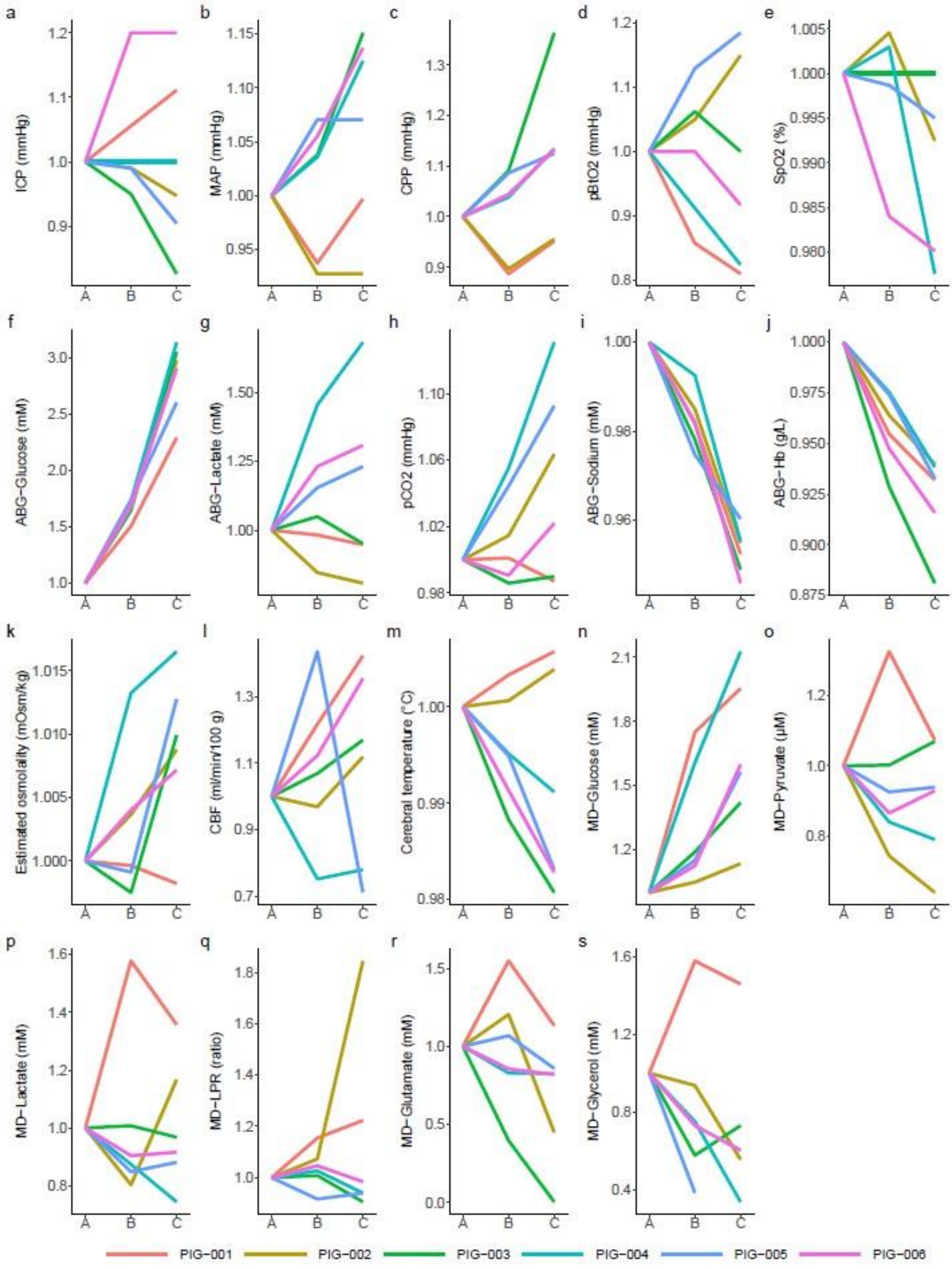

The figure illustrates the relative changes in the physiological variables following administration of moderate (*phase B*) and large (*phase C*) intravenous glucose in relation to baseline (*phase A*). As illustrated, intravenous glucose injections increased arterial glucose. The relative change in correlation coefficients of autoregulation, e.g., PRx and ORx, spanning from -1 to +1 was not considered meaningful. The lines in the figures are color-coded for each pig.

CBF = Cerebral blood flow. CPP = Cerebral perfusion pressure. ICP = Intracranial pressure. LPR = Lactate-/pyruvate ratio. MAP = Mean arterial blood pressure. MD = Microdialysis. $PbtO_2$ = Partial brain tissue oxygenation. PRx = Pressure reactivity index.

Supplementary figure 4. Cerebral autoregulation in the normal and injured brain state – TFA analysis

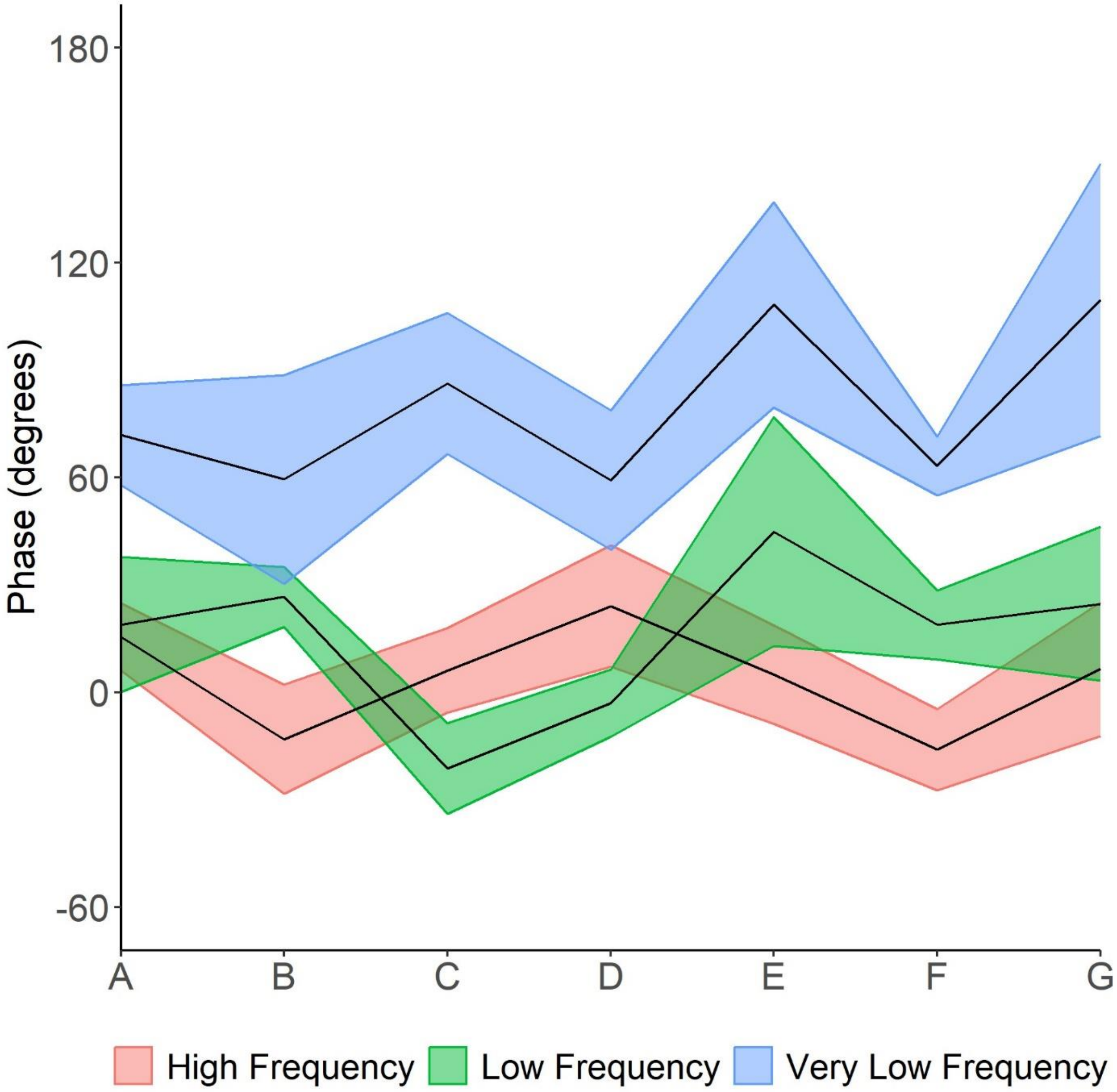

The figure illustrates the mean ± standard error of the phase shift for all of the experimental phases; baseline (A), moderate hyperglycemia (B), severe hyperglycemia (C), new baseline (D), ICP elevation (E), ICP elevation + severe hyperglycemia (F), and ICP elevation + insulin (G). As indicated, the phase shift was overall higher for the very low frequency ranges as compared to the low and high frequency ranges.

Supplementary figure 5. Cerebral autoregulation in the normal and injured brain state – TFA analysis including phase shift, coherence, and normalized gain

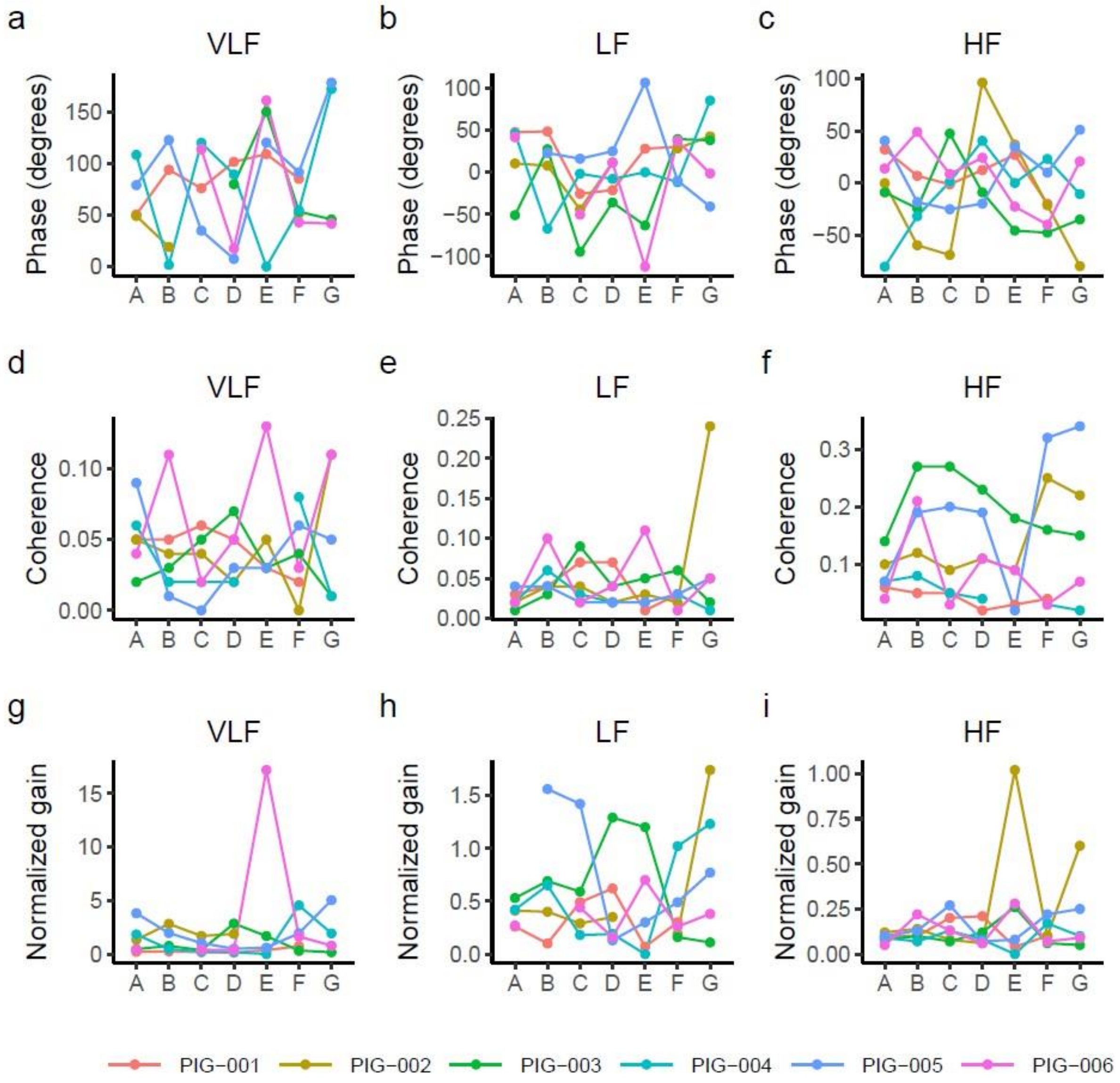

The figure illustrates the phase shift, coherence, and normalized gain for the very low, low, and high frequency ranges for all of the experimental phases; baseline (A), moderate hyperglycemia (B), severe hyperglycemia (C), new baseline (D), ICP elevation (E), ICP elevation + severe hyperglycemia (F), and ICP elevation + insulin (G).

Supplementary figure 6. Induction of intracranial hypertension, hyperglycemia, and restoration of arterial glucose in the injured brain state – a multimodality monitoring analyses

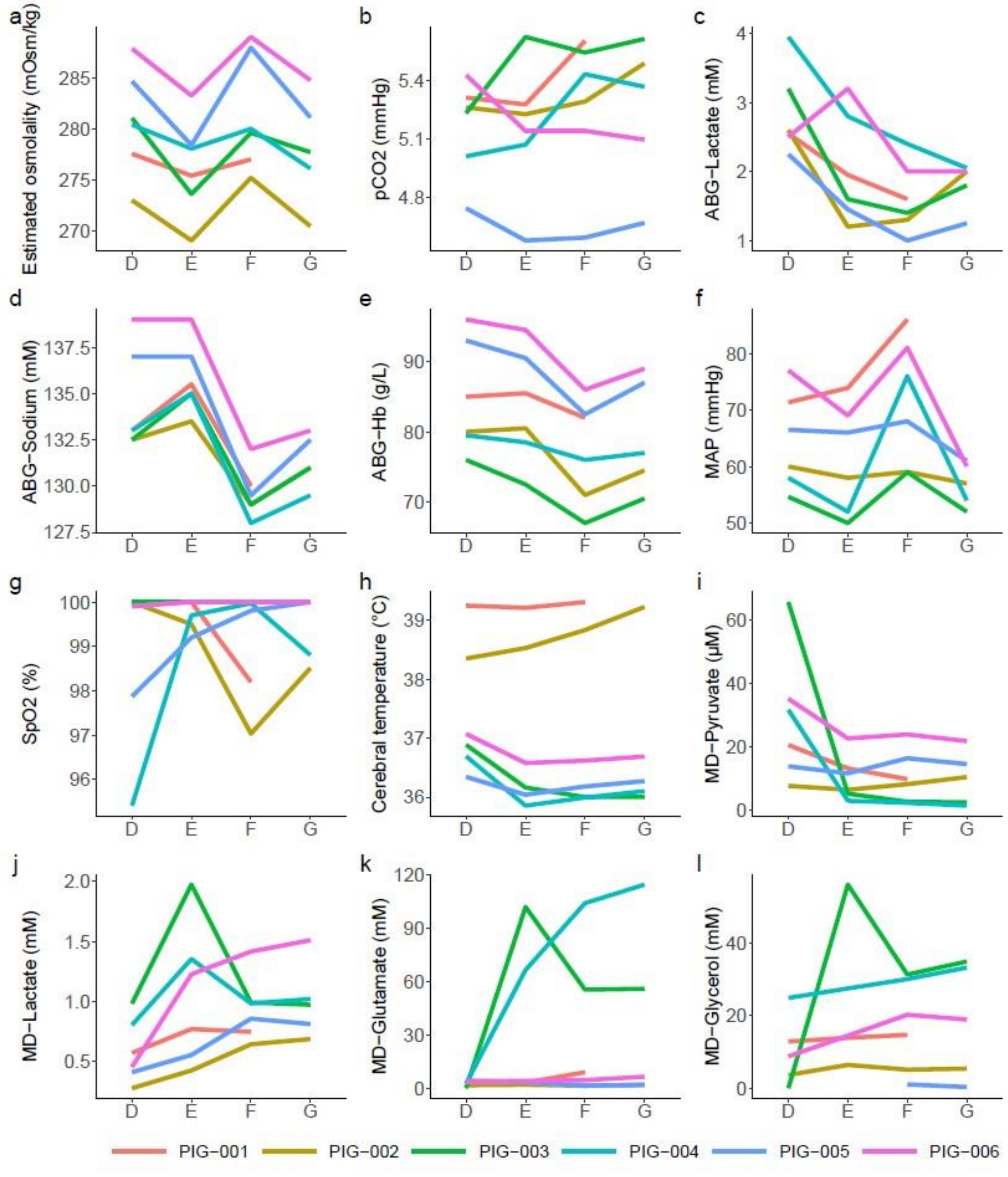

The figure illustrates the dynamics of the median values during each 30-minute episode of the re-established baseline (*phase D*), induction of intracranial hypertension with an intracranial, inflatable

balloon (*phase* E), administration of a large intravenous glucose injection (*phase* F), and restoration of arterial glucose with insulin (*phase* G) in six anesthetized pigs with invasive multimodality monitoring. As illustrated, intracranial hypertension compromised the brain state by lowering CPP, increasing PRx, decreasing CBF, $pbtO_2$, and MD-glucose, and increasing MD-LPR. Hyperglycemia (*phase* F*)* restored CBF and MD-glucose to some extent, while restoration of arterial glucose with insulin (*phase G*) had the opposite effect. The lines in the figures are color-coded for each pig.

CBF = Cerebral blood flow. CPP = Cerebral perfusion pressure. ICP = Intracranial pressure. LPR = Lactate-/pyruvate ratio. MAP = Mean arterial blood pressure. MD = Microdialysis. $PbtO_2$ = Partial brain tissue oxygenation. PRx = Pressure reactivity index.

Supplementary figure 7. Induction of intracranial hypertension, hyperglycemia, and restoration of arterial glucose in the injured brain state – a multimodality monitoring analyses – relative changes

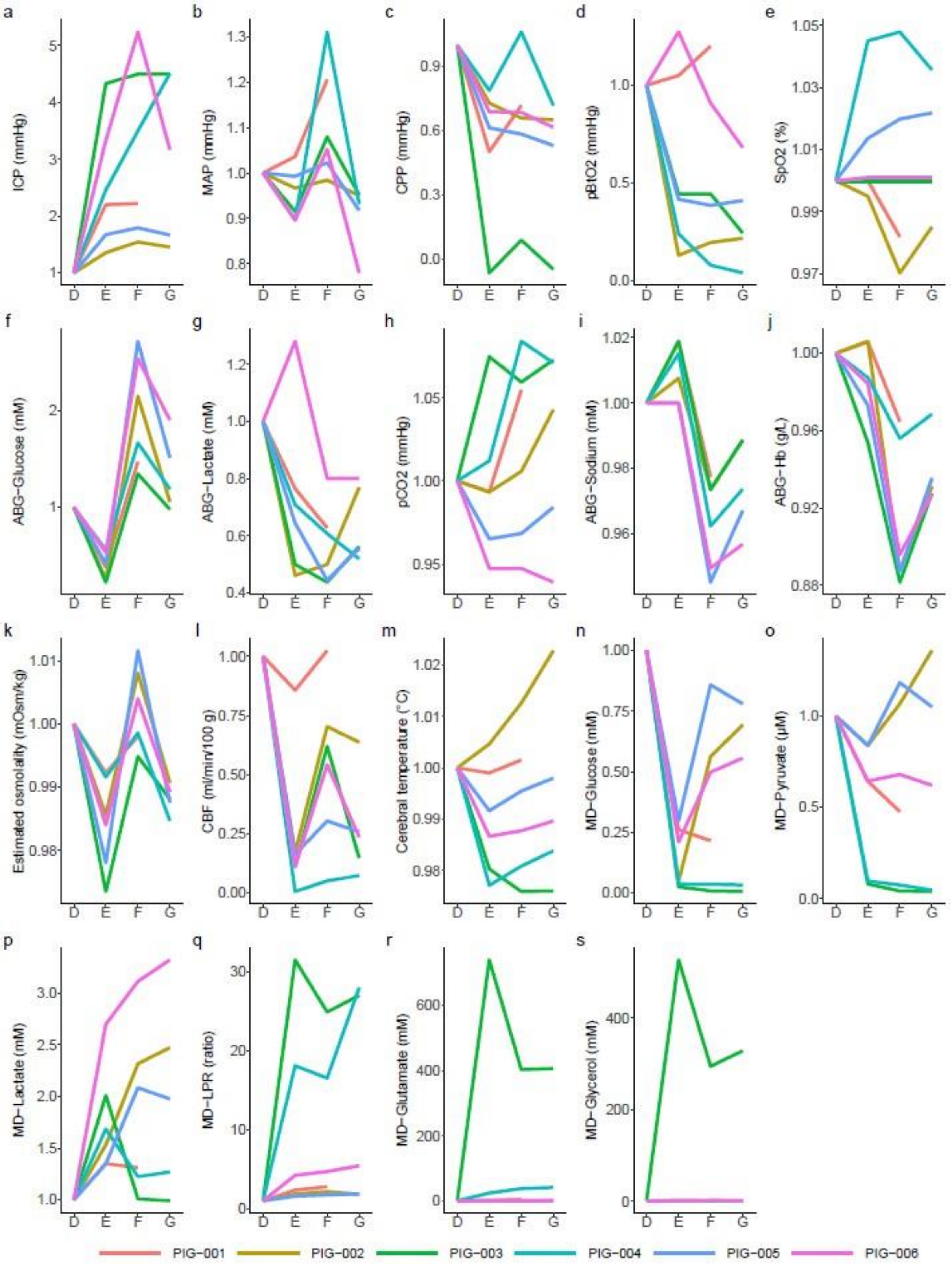

The figure illustrates the relative changes in the physiological variables following induction of intracranial hypertension (*phase E*), administration of a large intravenous glucose injection (*phase F*), and restoration of arterial glucose with insulin (*phase G*), in relation to baseline (*phase D*). The relative change in correlation coefficients of autoregulation, e.g., PRx and ORx, spanning from -1 to +1 was not considered meaningful. The lines in the figures are color-coded for each pig.

CBF = Cerebral blood flow. CPP = Cerebral perfusion pressure. ICP = Intracranial pressure. LPR = Lactate-/pyruvate ratio. MAP = Mean arterial blood pressure. MD = Microdialysis. $PbtO_2$ = Partial brain tissue oxygenation. PRx = Pressure reactivity index.
